# The PRESLO study: evaluation of a global secondary low back pain prevention program for health care personnel in a hospital setting. Multicenter, randomized intervention trial

**DOI:** 10.1186/1471-2474-13-234

**Published:** 2012-11-27

**Authors:** Angélique Denis, Amélie Zelmar, Marie-Annick Le Pogam, Emmanuelle Chaleat-Valayer, Alain Bergeret, Cyrille Colin

**Affiliations:** 1 Pôle Information Médicale Evaluation Recherche clinique, Hospices Civils de Lyon, Lyon, France; 2 Department of Physical and Rehabilitation Medicine, Hôpital de jour du Centre Médico-Chirurgical de Réadaptation des Massues Croix Rouge Française, Rouge, France; 3 Central Personnel Service for Occupational Medicine and Health Care, Centre Hospitalier Lyon Sud, Hospices Civils de Lyon, Lyon, France; 4 University of Lyon 1, Lyon, France

## Abstract

**Background:**

Common low back pain represents a major public health problem in terms of its direct cost to health care and its socio-economic repercussions. Ten percent of individuals who suffer from low back pain evolve toward a chronic case and as such are responsible for 75 to 80% of the direct cost of low back pain. It is therefore imperative to highlight the predictive factors of low back pain chronification in order to lighten the economic burden of low back pain-related invalidity. Despite being particularly affected by low back pain, Hospices Civils de Lyon (HCL) personnel have never been offered a specific, tailor-made treatment plan. The PRESLO study (with PRESLO referring to Secondary Low Back Pain Prevention, or in French, PREvention Secondaire de la LOmbalgie), proposed by HCL occupational health services and the Centre Médico-Chirurgical et de Réadaptation des Massues – Croix Rouge Française, is a randomized trial that aims to evaluate the feasibility and efficiency of a global secondary low back pain prevention program for the low back pain sufferers among HCL hospital personnel, a population at risk for recurrence and chronification. This program, which is based on the concept of physical retraining, employs a multidisciplinary approach uniting physical activity, cognitive education about low back pain and lumbopelvic morphotype analysis. No study targeting populations at risk for low back pain chronification has as yet evaluated the efficiency of lighter secondary prevention programs.

**Methods/Design:**

This study is a two-arm parallel randomized controlled trial proposed to all low back pain sufferers among HCL workers, included between October 2008 and July 2011 and followed over two years. The personnel following their usual treatment (control group) and those following the global prevention program in addition to their usual treatment (intervention group) are compared in terms of low back pain recurrence and the impairments measured at the beginning and the end of the study. The global prevention program is composed of a two-hour information session about low back pain and pain pathways, followed by five weekly 90-min exercise sessions with one physiotherapist per group of eight to ten personnel. A booklet for home use with patient-managed exercise instructions and information (The Back Book) is given to each participant at the end of the program.

An X-ray assessment of the entire spinal column of each participant (in both the control and intervention groups) is performed at the onset of the study in order to analyze sagittal spinopelvic balance as well as lombopelvic morphotype.

**Discussion:**

The results of this study, which is innovative and unique in France, will be available in 2014 and will make it possible to draw conclusions regarding the program’s impact on the risk of recurrence and chronification of low back pain.

**Trial registration:**

http://www.clinicaltrials.gov # NCT00782925

## Background

The estimated annual prevalence of low back pain is between 35 and 50% in France. As the third most important cause of chronic disability, low back pain is the chronic pathology that most often leads to activity limitations in persons aged 45 through 65 years old. Acute low back pain is a benign condition that heals within a few weeks in 90% of cases. That said, between 20 and 44% of patients undergo a recurrence within one year, and 5 to 10% of patients develop chronic low back pain, which in turn is responsible for 70 to 80% of the total cost of low back pain.

Hospital personnel are particularly affected by low back pain. At the Hospices Civils de Lyon (HCL), sick leave related to back problems represents the most important cause of work interruption.

The main risk factor for recurrence or chronification of low back pain is, however, a previous history of low back pain, which encompasses the concepts of severity, pain, duration and functional incapacity. It is therefore of utmost importance to provide treatment that is specifically targeted to these high-risk populations.

No specific medical care strategy has been implemented for these high-risk populations within hospital services. Training sessions focusing on the handling of patients and loads are currently offered to HCL personnel whether or not they suffer from low back pain. These training sessions are very general in nature and can be likened to a primary prevention strategy inspired by back schools. The efficiency of such training in terms of primary prevention has not been demonstrated. On the other hand, these sessions are offered to a population that already presents a history of low back pain, which is the main risk factor for recurrence and chronification.

Multidisciplinary physical retraining programs appeared in the 1980s. As opposed to back schools, these programs approach low back pain along its medico-psycho-social dimensions and take into account its multi-factorial origin. For dealing with chronic severe low back pain that has resisted conventional treatment, such programs are effective, especially in terms of returns to work. Due to their ponderous and expensive nature, however, these programs are reserved for chronic low back pain sufferers. Other programs based on physical retraining programs have since been developed. These lighter programs are now recommended to help prevent the chronification of less severe forms of low back pain.

PRESLO is a study proposed by the HCL occupational health service and the Centre Médico-Chirurgical et de Réadaptation des Massues – Croix Rouge Française that aims to evaluate the effectiveness of a global secondary low back pain prevention program for the HCL personnel who suffer from low back pain. Through its approach that takes into account the biomedical, environmental and psycho-behavioral factors of low back pain, this study provides a unique perspective.

This program, inspired by the concept of physical retraining, is based not only on an overall evaluation of physical, emotional and social dimensions, with for the biomedical angle the novel addition of a lumbopelvic morphotype analysis; but also on a multidisciplinary approach and care plan combining physical activity (training program followed by a home-based program) as well as cognitive learning on the topics of low back pain, pain pathways and the factors favoring the development of chronic low back pain.

## Method

### Objectives

#### Primary objective

To evaluate the effectiveness of a global secondary prevention program for acute or sub-acute low back pain in decreasing the recurrence of low back pain among health care professionals working in health care facilities.

#### Secondary objectives

The secondary objectives are: to evaluate the effectiveness of a global secondary prevention program for acute or sub-acute low back pain on the average time to recurrence of low back pain, and in decreasing the chronification of low back pain among professionals working in health care facilities; to identify risk factors for recurrence of low back pain (personal factors, psychosocial factors, sagittal spinopelvic balance); and, finally, to evaluate the feasibility of the global secondary back pain prevention program *via* the assessment of patient compliance to the program, the assessment of the program by the patients, and the evaluation of the operational model of the program by the patients and the health care professionals involved (physiotherapists and occupational health physicians).

### Study design

**Figure 1:**
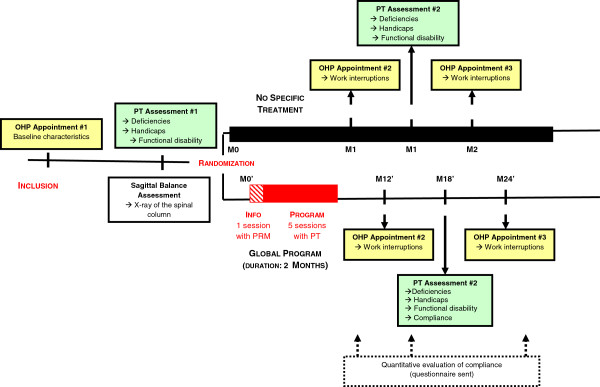
Organizational structure of the PRESLO study (OHP = Occupational Health Physician, PT = Physiotherapist, PRM = Physical and Rehabilitation Medicine Physician).

The study design as presented in Figure 1 is a two-arm parallel multicenter randomized controlled trial (usual treatment followed on the initiative of each participant versus treatment through the global prevention program).

### Participants and recruitment

The participating professionals were enrolled in the study by the occupational health physicians of the ten HCL hospital facilities involved, either during annual checkups or in the context of a spontaneous and voluntary process on the part of the professionals. Communication campaigns were regularly carried out in order to encourage inclusions (through an article in the HCL electronic newsletter, an information notice accompanying the salary statements of HCL professionals, and an article in the HCL’s specialized journals).

Inclusion and exclusion criteria

Inclusion Criteria

Were include the persons presenting all the following criteria:

Any member of HCL personnel regardless of occupation,

And presenting, over the past three years, one or more episodes of low back pain of less than three months’ duration, whether or not a work interruption was generated (low back pain, lumbo-sciatica or acute or sub-acute cruralgia are considered),

And consenting to participate in the study).

Exclusion Criteria

The persons presenting at least one of the following criteria were excluded:

Previous history of operated vertebral column fractures,

Previous history of discal hernia surgery in two or more locations,

Previous history of lumbar or lumbosacral arthrodesis in three or more locations,

Clinical presentation of radiculalgia with sequelary motor deficiencies or pure radiculalgia with positive Lasègue test (< 60°),

Indication for treatment in a functional restoration program for the vertebral column (that is, group III low back pain sufferers with permanent underlying pain for more than 3 months),

Current painful episode of acute lumbago,

Presence of psychiatric and/or behavioral disorders that decrease the reliability of evaluations,

Unstable cardiac pathology,

Insufficient mastery of the French language,

Pregnant woman.

### Randomization

Following the inclusion visit, the study group of the participants was decided by random allocation. The randomization was stratified and balanced by blocks between the participating occupational health services. The randomization lists were computed using SAS® statistical analysis software (Version 9.1, SAS Institute Inc., Cary, NC).

Each participant was informed by mail of his assignment to a study group following an entry checkup performed by a physiotherapist. In this letter, the study group was identified and, where appropriate, the timetable for the five global prevention program sessions was included. In order to ensure the objectivity of the evaluations, the occupational health physicians were not informed as to which study group each participant was assigned (single blind). At the time of enrollment, participants were also asked not to reveal their study group to the occupational health physician.

### Intervention

#### Intervention group: global prevention program

**Figure 2:**
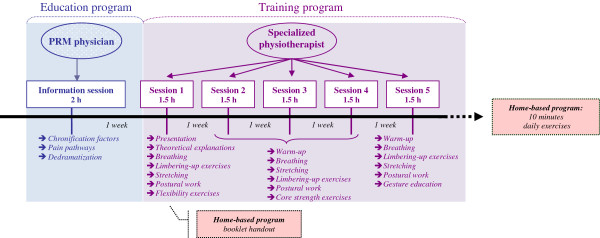
Procedure and content of the 6 sessions of the PRESLO global prevention program.

The global prevention program procedure is presented in Figure 2. This program requires approximately two months and is based on the management of low back pain on both the cognitive level (information about pain pathways and the factors favoring the development of chronification) and the physical level (implementation of an education and training program followed by a home-based program).

##### Education program

This low back pain information program is carried out at the start of the global program. It deals with the factors favoring chronification (including professional factors), the dedramatization of low back pain using anatomical explanations, pain pathways and the impact of emotional factors.

This roughly two-hour information session is led by a physician specialized in functional rehabilitation from the Centre Médico-Chirurgical et de Réadaptation des Massues – Croix Rouge Française, for a group of eight persons.

A copy of the information booklet entitled “Guide du dos,” the French version of The Back Book, is given to each participant at the end of the session as a source of complementary information. This booklet contains simple, reassuring advice about acute low back pain, in seven key messages. It has been demonstrated that the distribution of The Back Book decreases by 25% the number of patients suffering from persistent pain three months after an episode of acute low back pain [1]. Considering the validation and the impact of the messages disseminated in this booklet [2], [3] it appears relevant to use this information source in the context of the PRESLO study.

##### Training program

The training program involves five weekly sessions of 90 min each. The sessions are led by a physiotherapist from the Centre Médico-Chirurgical et de Réadaptation des Massues – Croix Rouge Française, in a rotating manner in order to limit the therapist effect. The patients meet in a group of eight to ten persons.

Each rehabilitation session is composed of three parts: first, a warm-up (15 min) with rhythmic exercises and changes in rhythm (walking forward, backward, on tiptoe, on heels, and so on); second, a period for stretching and mobilizing the rachis (60 min) including relaxation of the lumbar rachis and stretching of the hamstrings, gluteals, quadriceps, psoas and adductors, as well as pelvic tilt awareness exercises; and finally, a third part involving respiratory and postural work (15 min).

The program was conceived in a spirit of continuity and progression from one session to the next. A patient is therefore not allowed to pursue the program in case of absence (except for session 3 or session 4, which are very similar to each other, so one of these sessions can be substituted for the other).

The aims of the training program are: 1) to improve the patient’s knowledge and management of his back through practical education and theoretical explanations; 2) to lessen motor inhibition and the fear of movement through the use of simple, repeated exercises, in the aim of leading the participants toward awareness of their avoidance behavior and encouraging them to resume physical activity; 3) to reinforce global postural control and proprioceptive control; and 4) to raise awareness of factors that can modulate pain.

The physiotherapist emphasizes the purpose of each exercise and also suggests personalized exercises. The aim is to explain techniques and assist participants in their practical acquisition, so that the participants can learn to know and maintain their back.

The training sessions are carried out on the workplace site of the participants in a room that is appropriate for floor exercises.

##### Home-based program

A booklet of exercises is given to each participant at the end of the first session of the training program. The exercises in this home-based program are taught during the supervised training program sessions. The participants are instructed to perform the exercises on a daily basis for approximately ten minutes, at home, once the training program is finished.

#### Control group: usual treatment program

Control group members do not benefit from specific treatment of low back pain aside from any treatment undertaken on their own initiative (such as physiotherapy or mesotherapy). Information regarding the treatment program followed by each participant is collected by the occupational health physicians at the inclusion visit and during the follow-up visits.

### Implementation of the study

#### Baseline assessment

Baseline data (administrative, socio-demographic and socio-professional data, as well as information regarding the history of their condition) are collected by the occupational health physicians.

After his inclusion in the study, each participant, regardless of group, underwent an individual assessment with a physiotherapist from Centre Médico-Chirurgical et de Réadaptation des Massues – Croix Rouge Française, as well as an X-ray assessment of the spinal column. The clinical characteristics of the participants in terms of deficiencies, disability and handicap were collected during the initial assessment with the physiotherapist using clinical examination and self-administered questionnaires. The lumbopelvic morphotype of each participant was determined using the X-ray assessment of the entire spinal column carried out in the Radiology Department of the Hôpital Femme-Mère-Enfant (Groupement Hospitalier Est, Hospices Civils de Lyon).

#### Follow-up visits

All of the participants were followed for two years: either for the two years following the beginning of the global program for those in the intervention group, or for the two years following randomization for those in the control group. This monitoring was provided by the occupational health physician in the participant’s workplace (one appointment after 12 months and the other after 24 months) and by a physiotherapist from the Centre Médico-Chirurgical et de Réadaptation des Massues – Croix Rouge Française (one appointment after 18 months).

During the 12-month follow-up visit, the intervention group members participating in the evaluation of the operational model of the PRESLO study underwent a semi-structured interview with a member of the research team, immediately following the evaluation by the occupational health physician.

Participants’ presence at follow-up visits and sessions is continually verified using the attendance sheets transmitted by the physiotherapists. Participants who miss an assessment or an appointment are reminded by the Clinical Research Assistant from the data coordinating center concerning their participation in the study or questioned regarding their reasons for absence.

Outcomes

Primary outcome

Percentage of participants with at least one recurrence of low back pain in the two years following the end of the global program.

Secondary outcomes

1. Average time to recurrence of low back pain during the study period.

2. Percentage of participants with a sick leave related to a chronic low back pain (pain for more than three months) in the two years following the end of the global program.

3. Evaluation of the compliance of the participants in the global program.

4. Feedback from the participants in the global program.

5. Feedback from the health care professionals providing the program (physiotherapists and occupational health physicians).

A recurrence is defined as a new episode of low back pain leading to a sick leave. The occupational health physicians of each workplace have access to the date and duration of the work interruptions declared for each participant and can learn the motives from the participant at the annual checkups. The collection of information regarding low back pain recurrence will therefore be comprehensive.

#### Other parameters

##### Evaluation of deficiencies

Information relative to impairments is collected by the physiotherapist at the baseline and the 18-month follow-up visits.

Nature of the evaluation of impairments

1. Pain assessed using the short-form Saint-Antoine pain questionnaire [4] (QDSA)

2. Intensity of lumbar and radicular pains using a visual analog scale (in mm) between 0 (no pain) and 100 (pain of highest intensity)

3. Lumbar flexibility using the Schöber-MacRae test [5]

4. Greater and lesser pelvic flexibility using finger-ground distance

5. Hamstring flexibility using the thigh-to-leg angle (with coxofemoral joint at 90°)

6. Rectus femoris flexibility using the heel-to-buttock distance in prone position

7. Abdominal endurance (Shirado test) [6]

8. Lumbar extensor endurance (Sorensen test) [7,8]

9. Gluteal endurance (gluteals test)

10. Quadriceps endurance (Killy test)

##### Assessment of functional disabilities

Functional disability is assessed with the French version of the Quebec Back Pain Disability Scale [9,10], Its aim is to take into account the functional limitations linked to pain, in order to monitor the situation of low back pain sufferers enrolled in rehabilitation programs. An evaluation is planned at the baseline and the 18-month follow-up visits.

##### Assessment of lumbopelvic morphotype

An X-ray assessment of the entire spinal column is carried out at the start of the study for each participant by the Radiology Department of the Hôpital Femme-Mère-Enfant (Groupement Hospitalier Est, Hospices Civils de Lyon). This department is equipped with the EOS™ medical imaging system [11]. This machinery offers features that justify its use in a study of this type, such as considerable reduction in X-ray dose (eight to ten times less for 2D radiology, 800 to 1000 times less for 3D radiology), examination of the patient in a standing position allowing simultaneous front and lateral X-ray views from the top of the head to the soles of the feet, and the option of reconstructing every level of the osteo-articular system in 3D.

Two simultaneous views (front and lateral) are obtained from the top of the head (entire spinal column) to the tibial plateaus. The X-ray assessments are then analyzed using OPTISPINE® software (SMAIO Company) in cooperation with Centre Médico-Chirurgical et de Réadaptation des Massues – Croix Rouge Française, in order to determine the sagittal balance of each participant.

##### Assessment of handicap and functional impact

The arduousness of working conditions as well as job satisfaction are evaluated at the baseline and the 18-month follow-up visits using a numerical scale from 0 (not at all satisfied) to 10 (completely satisfied).

Anxio-depressive disorders are quantified at the baseline and 18-month follow-up visits with the Hospital and Anxiety Depression (HAD) scale [12].

The sensation of pain and the psychological experience of pain are evaluated at the baseline and the 18-month follow-up visit with the French version of the Fear Avoidance Beliefs Questionnaire (FABQ) [13-15]. These scores evaluate the fears, beliefs and attitude of the low back pain sufferer with respect to physical activity.

The coping strategies employed by the participants to address their pain are assessed at the baseline and the 18-month follow-up visits with the French version of the Coping Strategies Questionnaire (CSQ-F) (Rosenstiel and Keefe, 1983) [16,17]. Used in spinal pathology [18,19], this questionnaire permits the assessment of the psychological adaptation strategies used by patients faced with a pain-inducing stressor.

##### Assessment of quality of life

The quality of life of the participants is evaluated by a physiotherapist at the baseline and the 18-month follow-up visit with the SF-12 self-administered questionnaire, which is a short version of the Medical Outcomes Study Short-Form General Health Survey (SF-36). It allows the measurement of eight aspects of quality of life: general and mental health, physical and social functioning, physical and “emotional” health, pain and vitality [20].

##### Assessment of compliance with the global program

Compliance with the home-based program exercises is measured using two different approaches.

The first approach, quantitative evaluation, refers to the feedback gathered 6, 12 and 24 months after the end of the global program in terms of the number of different exercises performed, the number of weekly sessions and the weekly duration of exercises. A questionnaire is sent to the participants each time.

The second approach, qualitative evaluation, considers the quality of execution of the exercises. In this case, compliance is evaluated by the physiotherapist during the 18-month follow-up visit using a five-class semi-quantitative ordinal scale (null, poor, average, good and perfect).

##### Assessment of the global program by the participant

Each participant provides feedback on the global program using a five-class semi-quantitative ordinal scale (very satisfied, somewhat satisfied, neutral, somewhat unsatisfied and very unsatisfied). A questionnaire is sent to the participants 6, 12 and 24 months after the end of the global program in order to evaluate general satisfaction with the program, with the physical exercises, with the messages and information provided, with general program organization and with the program’s impact on health.

##### Assessment of the operational model

Each participant among the thirty or so recruited for this portion of the study will relate his experience of the program during the individual interviews. The experience of the occupational health physicians and of the physiotherapists will also be collected through discussion groups. The program’s elements of satisfaction/dissatisfaction as well as feasibility/acceptability will be studied using the thematic analysis of the content of these interviews and discussion groups.

**Table 1:** Management of protocol violations

**Type of protocol violation**	**Definition**
1. Erroneous inclusions	
2. Poor compliance with the global program sessions	Only one absence, to either session 3 or session 4 of the training program, is tolerated from among the 6 sessions of the global program. In other cases, participants are invited to make up the sessions with another group, in order to limit “program withdrawals.” In the case of an incomplete program, the participants in the intervention group are considered to be non-compliant and deviating from the protocol.
3. Non-attendance at follow-up visits	Participants who do not attend the annual checkups with the occupational health physician of their workplace or the final physiotherapy assessment are first reminded by mail, then by telephone if necessary. It is important to have access to main criterion data for all participants in order to proceed to intention-to-treat analysis.
4. Early withdrawal	This can take place by decision of the participant or following the occurrence of an adverse event that calls into question his participation in the study. Withdrawn participants are not replaced.
5. Missing data concerning the primary outcome (sick leave related to low back pain episode)	These participants cannot be taken into account in the analysis of data.

### Analysis

Data analysis will be carried out with SAS® statistical analysis software (Version 9.2, SAS Institute Inc., Cary, USA). Results will be expressed with their 95% confidence intervals. All statistical tests were two sided p-value less than 0.05 regarded as significant. An intention-to-treat analysis will first be performed on all of the participants. This analysis may be supplemented with a per-protocol analysis involving the participants for whom the follow-up adhered to the protocol, therefore excluding all deviations from the protocol (see above Table 1).

### Number of subjects required

With a recurrence rate estimated at 45% for the general population, in order to observe a 25% reduction in recurrence rate that is attributable to the global secondary prevention program, 310 participants per group (a total of 620 participants) are required to achieve at 80% power with Type 1 error rate of 5%.

A 5% increase in the number of participants is planned in order to take into account those lost to follow-up who do not attend all appointments, as well as any early dropouts. The total number of subjects is therefore set at 650.

These 650 professionals were recruited from within ten occupational health services belonging to the participating hospital facilities.

### Descriptive analysis

Baseline characteristics (socio-demographic, socio-professional and history of the disease) will be summarized and compared in the 2 study groups with the use of chi-square tests for categorical variables and two-sample t-tests or Wilcoxon’s test, where appropriate, for continuous variables, to ensure that participants in both groups were similar at the entry of study.

### Analysis of the primary outcome

The percentage of patients with at least one recurrent episode of low back pain during the 2-year follow-up will be compared between the 2 study groups using a chi-squared test. The prognostic factors and the possible interactions between covariables will be tested with the use of Mantel-Haenszel chi-squared analysis. A multivariate logistic regression analysis will be performed to evaluate the effectiveness of the global program after controlling for potential confounding variables.

### Analysis of the secondary outcomes

The effectiveness of the global program on the average time to recurrence of low back pain will be tested with the use of the Kaplan-Meier method and the logrank test. A multivariate Cox regression analysis will also be performed to adjust for the global program and all potential confounding variables.

The percentage of participants whose low back pain has become chronic in the two years following the program will be compared between the 2 study groups using a chi-squared test.

Qualitative thematic analysis of the content of the perceptions of the program users and of the health care professionals providing the program in terms of its organizational arrangements

This will be a qualitative thematic analysis of the content that aims to identify the elements of satisfaction or dissatisfaction and the degree of acceptability of the program for the various protagonists. These elements will be coded using the categories used initially in the interview guides (deductive approach). New coding categories will be generated through an inductive approach for the topics that do not correspond to the categories laid out in the interview guides. The coding will be carried out using Atlas.ti v5.2 qualitative analysis software [21].

### Participation time in the study

Participation time in the study is counted as work time for the participants. Participation time outside of working hours is therefore credited to each working hours account. This procedure does not modify the schedule of the departments concerned, which facilitates the implementation of the study within the health care services as well as the compliance of the participants. For the participation time taken during working hours, the reimbursement of these hours to the HCL is incorporated into the study budget. The participation time of each member of personnel requires precise counting and monitoring *via* the attendance sheets that are filled in and signed at every assessment and session, each time with the specification of whether or not working hours are involved.

### Ethics and registration

As the promoter of this biomedical research, which falls within the scope of French Law n°2004-806 of 9 August 2004, the Hospices Civils de Lyon have acquired liability insurance coverage and stand as guarantors of the proper execution of the study (monitoring and management of serious adverse events and on-site monitoring visits).

The study was approved by the relevant authorities (Ethics Committee, Directorate-General for Health and Consumers, National Commission for Data Protection and Liberties – CNIL France).

Approval was also given by the management of participating institutions as well as by the corresponding Committees for Health, Safety and Working Conditions (CHSCT).

### Benefits

The PRESLO study presents four major individual and collective advantages: 1) that this program could be complementary to the training sessions focusing on handling (of patients and/or of loads) that are currently offered to all HCL personnel whether or not they suffer from low back pain. These very general training sessions can be likened to a primary prevention strategy and are therefore insufficient or even useless for low back pain sufferers. It is therefore of utmost importance to offer treatment that is targeted specifically to these populations at risk for recurrence and chronification. The PRESLO program is particularly valuable in that it is open also to medico-technical and administrative personnel to whom these handling sessions are not relevant and therefore not offered; 2) that the necessary dialogue between hospital personnel with low back pain and their physicians should find itself stimulated on both preventive and curative levels; 3) that through the identification of the risk factors for recurrence and chronification of low back pain it should in time become possible to offer early, appropriate treatments for the prevention of chronic low back pain; and 4) that the direct costs (medicinal and medical/paramedical treatment) and indirect costs (sick leave and work absence, invalidity) related to low back pain should decrease over time.

If the efficiency of such a secondary prevention program is demonstrated at the end of the study, it could initially be perpetuated among the training sessions offered by the Hospices Civils de Lyon to their workers, and later implemented in other hospitals or health care institutions. This program could also be adapted and tested for professional sectors other than the hospital environment, since it is not specific to hospital personnel and could be offered *a priori* to all low back pain sufferers regardless of their background.

## Discussion

The purpose of this randomized study is to demonstrate the impact of a global secondary low back pain prevention program on a decrease in low back pain recurrence among personnel working in a health care institution. The high prevalence of low back pain, the high costs associated with this condition as well as the alteration of the quality of life of low back pain sufferers make this a study of strong scientific, economic and social value. Its study population and its methodology contribute to its unique, innovative character in France. Indeed, this is the first study to examine the secondary prevention of low back pain in a large population of health care institution personnel in France. The PRESLO program aims to place each member of the Hospices Civils de Lyon personnel who suffers from acute or sub-acute low back pain at the center of a preventive approach. The purpose is to facilitate the active involvement of the patient in his own care and treatment. Through PRESLO, the participant will learn to identify his own avoidance strategies and erroneous beliefs and to manage his pain on a daily basis using physical and respiratory exercises learned during the program sessions. As opposed to rest, which is contraindicated, this active approach will allow the participant to pursue or resume physical activity through pain. The risk of low back pain recurrence and the associated sick leaves should therefore decrease. An approach that is both quantitative and qualitative will be used to verify these assumptions. A qualitative analysis of the program’s operational model will be carried out using the perception of the program held by the participating personnel and by the physiotherapists and PRM physicians providing the program. The program’s users will therefore have the opportunity to express themselves regarding the treatment received in terms of its content and its organization. This procedure could in time lead to measures to improve the program, which could only increase the feasibility of the program, not only for its users, but also for its providers. The results of this qualitative analysis will enrich those of the quantitative analyses, particularly if clinical benefit is not demonstrated at the end of the study. Finally, the sagittal balance analysis of the personnel with low back pain is another original component of this study. It is possible that the risk of spinal pathologies may differ according to the back type of the patient. It therefore seems important to consider the sagittal balance evaluation based on X-ray views on the same level as the evaluation of the psycho-social and emotional dimension, when analyzing the case of a patient with low back pain. The results of the sagittal balance analysis will, moreover, be the first lombopelvic morphotype data yet published relative to a population of individuals with acute and sub-acute low back pain.

From a methodological viewpoint, however, the absence of a double-blind design and of a placebo control group could introduce bias into the interpretation of the results. That said, the very nature of the evaluated intervention did not allow such a design. Cluster randomized trials, with departments randomized instead of individuals, could also have been chosen in order to alleviate non-blinded bias and the non-negligible risk of contamination between the two study groups. This possibility could nevertheless not be retained due to the diverse nature of the participants’ departments of origin. The proposed study is therefore an open randomized trial. The primary outcome, which is low back pain recurrence as quantified by related work absence is, however, an objective criterion. Also, participants in the intervention group were requested not to discuss the global secondary prevention program with the other participants. As for the potential loss of opportunity for the participants randomized into the control group, it was partially resolved through offering these participants the chance to take part in the information session of the global program following their 24-month monitoring period.

It is possible that some participants may concurrently take part in the training sessions dealing with the handling of patients or loads that the Hospices Civils de Lyon offer to all personnel whether or not they suffer from low back pain. These sessions are more general than the global program and should instead be considered as a primary prevention strategy. They are therefore not specifically oriented toward the treatment of low back pain and its recurrences. Their efficiency in terms of secondary prevention has never been demonstrated. Information regarding the participants’ possible involvement in these training sessions will be collected and taken into account in the analysis of data. In particular, the comparability of this criterion will be verified for the two groups.

Inclusions for this study began in October 2008. The results concerning the program’s impact on low back pain recurrence risk will be known during the first semester of 2014, since the follow-up of the last participants included is to reach conclusion at the end of 2013.

## Competing interests

The authors declare that they have no competing interests.

## Authors’ contributions

All authors participated in the design of the study and drafting the manuscript. All authors read and approved the final manuscript.

## Pre-publication history

The pre-publication history for this paper can be accessed here:

http://www.biomedcentral.com/1471-2474/13/234/prepub
